# *N*-(4-Bromo­phen­yl)-2,6-dimethyl-1,3-dioxan-4-amine

**DOI:** 10.1107/S1600536813025750

**Published:** 2013-09-21

**Authors:** Zeenat Fatima, Gottimukkala Rambabu, Bandapalli Palakshi Reddy, Vijayaparthasarathi Vijayakumar, Devadasan Velmurugan

**Affiliations:** a Centre of Advanced Study in Crystallography and Biophysics, University of Madras, Guindy Campus, Chennai 600 025, India; b Chemistry Department, GEBH, Sree Vidyanikethan Engineering College, A. Rangampet, Tirupati 517102, India; c Centre for Organic and Medicinal Chemistry, VIT University, Vellore 632 014, India

## Abstract

In the title compound, C_12_H_16_BrNO_2_, the dioxane ring adopts a chair conformation and its mean plane makes a dihedral angle of 60.63 (12)° with the 4-bromo­phenyl ring. In the crystal, mol­ecules are linked by pairs of N—H⋯O hydrogen bonds, forming inversion dimers with an *R*_2_^2^(8) ring motif. These dimers are consolidated by pairs of C—H⋯O hydrogen bonds with an *R*_2_^2^(16) ring motif. Adjacent dimers are connected *via* C—H⋯O hydrogen bonds, forming infinite chains propagating along the *c-*axis direction.

## Related literature

For biological properties of dioxanes and applications of 1,3-dioxane derivatives, see: Aubele *et al.* (2005 ▶); Marucci *et al.* (2005 ▶); Wang *et al.* (1996*a* ▶,*b* ▶); Yuan *et al.* (2005 ▶). For related crystal structures, see: Chuprunov *et al.* (1981 ▶); Thevenet *et al.* (2010 ▶). For hydrogen-bond motifs, see: Bernstein *et al.* (1995 ▶).
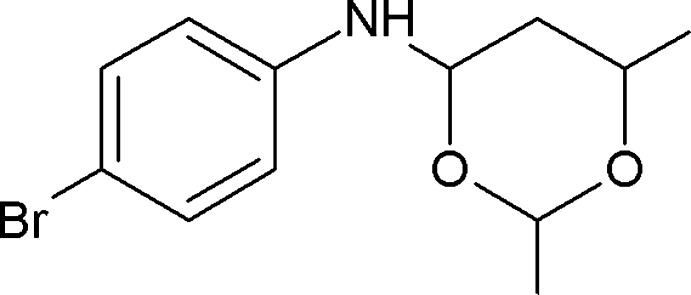


## Experimental

### 

#### Crystal data

C_12_H_16_BrNO_2_
*M* *_r_* = 286.17
Monoclinic, 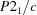

*a* = 9.9367 (5) Å
*b* = 13.5660 (6) Å
*c* = 10.3206 (5) Å
β = 115.543 (3)°
*V* = 1255.25 (10) Å^3^
*Z* = 4
Mo *K*α radiation
μ = 3.26 mm^−1^
*T* = 293 K
0.25 × 0.20 × 0.15 mm

#### Data collection

Bruker SMART APEXII area-detector diffractometer
Absorption correction: multi-scan (*SADABS*; Bruker, 2008 ▶) *T*_min_ = 0.310, *T*_max_ = 0.746
11981 measured reflections
3119 independent reflections
1819 reflections with *I* > 2σ(*I*)
*R*_int_ = 0.032

#### Refinement

*R*[*F* ^2^ > 2σ(*F* ^2^)] = 0.037
*wR*(*F* ^2^) = 0.092
*S* = 1.01
3119 reflections
150 parameters
1 restraint
H atoms treated by a mixture of independent and constrained refinement
Δρ_max_ = 0.45 e Å^−3^
Δρ_min_ = −0.38 e Å^−3^

### 

Data collection: *APEX2* (Bruker, 2008 ▶); cell refinement: *SAINT* (Bruker, 2008 ▶); data reduction: *SAINT*; program(s) used to solve structure: *SHELXS97* (Sheldrick, 2008 ▶); program(s) used to refine structure: *SHELXL97* (Sheldrick, 2008 ▶); molecular graphics: *ORTEP-3 for Windows* (Farrugia, 2012 ▶); software used to prepare material for publication: *SHELXL97* and *PLATON* (Spek, 2009 ▶).

## Supplementary Material

Crystal structure: contains datablock(s) global, I. DOI: 10.1107/S1600536813025750/su2646sup1.cif

Structure factors: contains datablock(s) I. DOI: 10.1107/S1600536813025750/su2646Isup2.hkl

Supplementary material file. DOI: 10.1107/S1600536813025750/su2646Isup3.cml

Additional supplementary materials:  crystallographic information; 3D view; checkCIF report

## Figures and Tables

**Table 1:** Hydrogen-bond geometry (Å, °)

*D*—H⋯*A*	*D*—H	H⋯*A*	*D*⋯*A*	*D*—H⋯*A*
N1—H1⋯O1^i^	0.82 (2)	2.66 (2)	3.465 (2)	168 (2)
C8—H8⋯O2^i^	0.93	2.51	3.352 (3)	150
C9—H9⋯O2^ii^	0.93	2.65	3.557 (3)	165

Symmetry codes: (i) 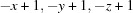; (ii) 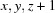.

**Fig. 1.**
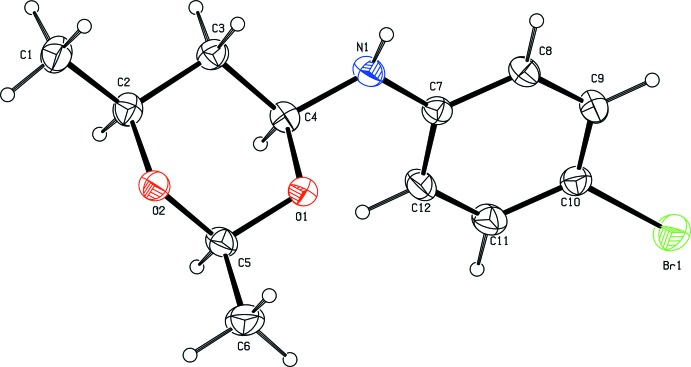
The molecular structure of the title molecule, with atom labelling. Displacement ellipsoids are drawn at the 30% probability level.

**Fig. 2.**
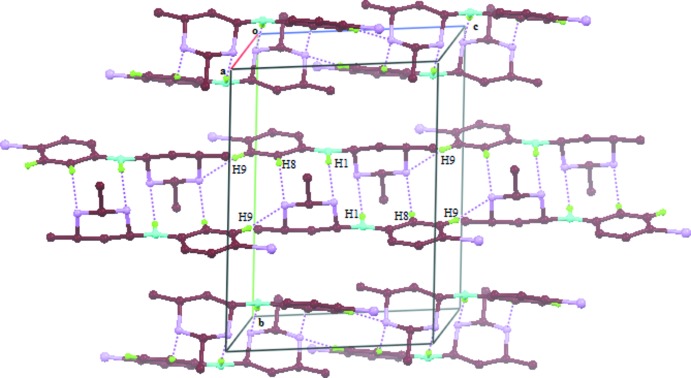
The crystal packing of the title compound viewed along the a axis. The hydrogen bonds are shown as dashed lines (see Table 1 fro details; H atoms not involved in hydrogen bonding have been omitted for clarity).

## supplementary crystallographic information

### 1. Comment

Dioxane rings are frequently encountered as structural motifs in many bioactive molecules such as cytotoxic agents (Aubele *et al.*, 2005) and antimuscarinic agents (Marucci *et al.*, 2005). 1,3-dioxane derivatives have applications in fine medicinal chemistry in the pharmaceutical (Wang *et al.*, 1996*a*) and cosmetic industries (Wang *et al.*, 1996*b*; Yuan *et al.*, 2005). In view of the excellent biological and pharmacological applications of this class of compounds, we report herein on the synthesis and crystal structure of the title compound.

In the title compound, Fig 1, the dioxane ring (O1/O2/C2—C5) adopts a chair conformation and its mean plane makes a dihedral angle of 60.63 (12)° with the benzene ring (C7—C12).

In the crystal, molecules are linked by a pair of N—H···O hydrogen bonds forming inversion dimers with an *R*_2_^2^(8) ring motif (Bernstein *et al.*, 1995). These dimers are consolidated by a pair of C—H···O hydrogen bonds with an *R*_2_^2^(16) ring motif. Adjacent dimers are connected *via* C—H···O hydrogen bonds forming infinite chains propagating along the *c* axis (see Table 1 and Fig. 2 for details).

### 2. Experimental

To 4-bromoaniline (1 mmol), acetaldehyde (3 mmol) was added drop wise and stirred for about 4 h at 273 K. The progress of the reaction was monitored through TLC. On completion of the reaction the mixture was washed with petroleum ether. The resultant product was dissolved in diethylether and allowed to evaporate. Block-like colourless crystals of the title compound were obtained by recrystallization with diethylether.

### 3. Refinement

The NH H atom was refined with a distance restraint of N-H = 0.86 (2) Å with U_iso_(H) = 1.2U_eq_(N). The C bound H atoms were placed in calculated positions and refined as riding atoms: C-H = 0.93 - 0.98 Å with *U*_iso_(H) = 1.5*U*_eq_(C-methyl) and = 1.2*U*_eq_(C) for other H atoms.

### Crystal data

**Table:**

C_12_H_16_BrNO_2_	*F*(000) = 584
*M**_r_* = 286.17	*D*_x_ = 1.514 Mg m^−^^3^
Monoclinic, *P*2_1_/*c*	Mo *K*α radiation, λ = 0.71073 Å
Hall symbol: -P 2ybc	Cell parameters from 3119 reflections
*a* = 9.9367 (5) Å	θ = 2.3–28.4°
*b* = 13.5660 (6) Å	µ = 3.26 mm^−^^1^
*c* = 10.3206 (5) Å	*T* = 293 K
β = 115.543 (3)°	Block, colourless
*V* = 1255.25 (10) Å^3^	0.25 × 0.20 × 0.15 mm
*Z* = 4	

### Data collection

**Table:**

Bruker SMART APEXII area-detector diffractometer	3119 independent reflections
Radiation source: fine-focus sealed tube	1819 reflections with *I* > 2σ(*I*)
Graphite monochromator	*R*_int_ = 0.032
ω and φ scans	θ_max_ = 28.4°, θ_min_ = 2.3°
Absorption correction: multi-scan (*SADABS*; Bruker, 2008)	*h* = −12→13
*T*_min_ = 0.310, *T*_max_ = 0.746	*k* = −18→15
11981 measured reflections	*l* = −13→13

### Refinement

**Table:**

Refinement on *F*^2^	Primary atom site location: structure-invariant direct methods
Least-squares matrix: full	Secondary atom site location: difference Fourier map
*R*[*F*^2^ > 2σ(*F*^2^)] = 0.037	Hydrogen site location: inferred from neighbouring sites
*wR*(*F*^2^) = 0.092	H atoms treated by a mixture of independent and constrained refinement
*S* = 1.01	*w* = 1/[σ^2^(*F*_o_^2^) + (0.0432*P*)^2^ + 0.0602*P*] where *P* = (*F*_o_^2^ + 2*F*_c_^2^)/3
3119 reflections	(Δ/σ)_max_ = 0.001
150 parameters	Δρ_max_ = 0.45 e Å^−^^3^
1 restraint	Δρ_min_ = −0.38 e Å^−^^3^

### Special details

**Table:**

Geometry. All e.s.d.'s (except the e.s.d. in the dihedral angle between two l.s. planes) are estimated using the full covariance matrix. The cell e.s.d.'s are taken into account individually in the estimation of e.s.d.'s in distances, angles and torsion angles; correlations between e.s.d.'s in cell parameters are only used when they are defined by crystal symmetry. An approximate (isotropic) treatment of cell e.s.d.'s is used for estimating e.s.d.'s involving l.s. planes.
Refinement. Refinement of *F*^2^ against ALL reflections. The weighted *R*-factor *wR* and goodness of fit *S* are based on *F*^2^, conventional *R*-factors *R* are based on *F*, with *F* set to zero for negative *F*^2^. The threshold expression of *F*^2^ > σ(*F*^2^) is used only for calculating *R*-factors(gt) *etc*. and is not relevant to the choice of reflections for refinement. *R*-factors based on *F*^2^ are statistically about twice as large as those based on *F*, and *R*- factors based on ALL data will be even larger.

### Fractional atomic coordinates and isotropic or equivalent isotropic displacement parameters (Å^2^)

**Table:**

	*x*	*y*	*z*	*U*_iso_*/*U*_eq_	
Br1	1.07805 (3)	0.63232 (2)	1.22087 (3)	0.07742 (15)	
O1	0.70096 (17)	0.52265 (9)	0.47016 (15)	0.0474 (4)	
O2	0.61959 (17)	0.53559 (10)	0.22317 (15)	0.0488 (4)	
N1	0.6108 (2)	0.62162 (13)	0.6010 (2)	0.0502 (5)	
H1	0.531 (2)	0.5954 (17)	0.588 (3)	0.060*	
C1	0.4240 (3)	0.64080 (17)	0.0718 (3)	0.0640 (7)	
H1A	0.4566	0.6270	−0.0014	0.096*	
H1B	0.3852	0.7067	0.0598	0.096*	
H1C	0.3475	0.5949	0.0642	0.096*	
C2	0.5537 (3)	0.63121 (15)	0.2171 (2)	0.0497 (6)	
H2	0.6276	0.6818	0.2260	0.060*	
C3	0.5121 (3)	0.64209 (15)	0.3411 (3)	0.0504 (6)	
H3A	0.4781	0.7088	0.3433	0.060*	
H3B	0.4311	0.5973	0.3282	0.060*	
C4	0.6445 (3)	0.61968 (14)	0.4810 (2)	0.0461 (5)	
H4	0.7225	0.6685	0.4960	0.055*	
C5	0.7436 (3)	0.51969 (17)	0.3564 (2)	0.0522 (6)	
H5	0.8196	0.5701	0.3714	0.063*	
C6	0.8065 (3)	0.4193 (2)	0.3537 (3)	0.0756 (8)	
H6A	0.8360	0.4162	0.2766	0.113*	
H6B	0.7320	0.3701	0.3394	0.113*	
H6C	0.8917	0.4075	0.4432	0.113*	
C7	0.7211 (3)	0.62384 (13)	0.7422 (2)	0.0433 (5)	
C8	0.6828 (3)	0.60493 (15)	0.8537 (3)	0.0501 (6)	
H8	0.5844	0.5896	0.8328	0.060*	
C9	0.7872 (3)	0.60831 (15)	0.9954 (3)	0.0545 (6)	
H9	0.7594	0.5944	1.0687	0.065*	
C10	0.9320 (3)	0.63231 (14)	1.0272 (3)	0.0504 (6)	
C11	0.9719 (3)	0.65323 (19)	0.9185 (3)	0.0622 (7)	
H11	1.0698	0.6707	0.9402	0.075*	
C12	0.8674 (3)	0.64851 (18)	0.7771 (3)	0.0586 (6)	
H12	0.8960	0.6622	0.7042	0.070*	

### Atomic displacement parameters (Å^2^)

**Table:**

	*U*^11^	*U*^22^	*U*^33^	*U*^12^	*U*^13^	*U*^23^
Br1	0.0657 (2)	0.1039 (3)	0.05105 (18)	0.01315 (15)	0.01413 (14)	−0.00249 (14)
O1	0.0466 (10)	0.0523 (8)	0.0450 (8)	0.0057 (7)	0.0215 (7)	0.0021 (7)
O2	0.0433 (9)	0.0577 (9)	0.0437 (9)	−0.0010 (7)	0.0170 (7)	−0.0041 (7)
N1	0.0444 (12)	0.0600 (12)	0.0494 (11)	−0.0045 (9)	0.0232 (10)	−0.0055 (9)
C1	0.0644 (18)	0.0693 (16)	0.0515 (15)	0.0022 (13)	0.0185 (13)	0.0100 (12)
C2	0.0489 (15)	0.0482 (13)	0.0496 (13)	−0.0078 (10)	0.0189 (12)	0.0034 (10)
C3	0.0513 (15)	0.0458 (12)	0.0521 (14)	0.0051 (11)	0.0205 (12)	0.0013 (10)
C4	0.0472 (14)	0.0483 (12)	0.0449 (12)	−0.0050 (10)	0.0218 (11)	−0.0036 (10)
C5	0.0410 (14)	0.0714 (15)	0.0454 (13)	−0.0020 (11)	0.0199 (11)	−0.0053 (11)
C6	0.067 (2)	0.0956 (19)	0.0612 (17)	0.0282 (16)	0.0250 (15)	−0.0049 (15)
C7	0.0451 (14)	0.0386 (11)	0.0480 (12)	0.0019 (10)	0.0219 (11)	−0.0023 (9)
C8	0.0457 (15)	0.0546 (12)	0.0556 (14)	−0.0051 (11)	0.0273 (12)	−0.0005 (11)
C9	0.0614 (18)	0.0605 (14)	0.0499 (13)	0.0015 (12)	0.0319 (13)	0.0046 (11)
C10	0.0494 (15)	0.0528 (13)	0.0471 (13)	0.0079 (11)	0.0190 (11)	−0.0007 (10)
C11	0.0418 (15)	0.0861 (17)	0.0596 (16)	−0.0025 (12)	0.0226 (13)	−0.0010 (13)
C12	0.0468 (15)	0.0854 (17)	0.0514 (15)	−0.0032 (13)	0.0284 (12)	0.0014 (12)

### Geometric parameters (Å, º)

**Table:**

Br1—C10	1.898 (2)	C4—H4	0.9800
O1—C5	1.410 (2)	C5—C6	1.504 (3)
O1—C4	1.454 (2)	C5—H5	0.9800
O2—C5	1.412 (3)	C6—H6A	0.9600
O2—C2	1.442 (2)	C6—H6B	0.9600
N1—C7	1.396 (3)	C6—H6C	0.9600
N1—C4	1.416 (3)	C7—C12	1.379 (3)
N1—H1	0.824 (16)	C7—C8	1.383 (3)
C1—C2	1.503 (3)	C8—C9	1.382 (3)
C1—H1A	0.9600	C8—H8	0.9300
C1—H1B	0.9600	C9—C10	1.369 (4)
C1—H1C	0.9600	C9—H9	0.9300
C2—C3	1.513 (3)	C10—C11	1.371 (3)
C2—H2	0.9800	C11—C12	1.381 (4)
C3—C4	1.507 (3)	C11—H11	0.9300
C3—H3A	0.9700	C12—H12	0.9300
C3—H3B	0.9700		
			
C5—O1—C4	110.76 (15)	O1—C5—C6	108.55 (19)
C5—O2—C2	111.66 (16)	O2—C5—C6	108.37 (19)
C7—N1—C4	122.5 (2)	O1—C5—H5	109.7
C7—N1—H1	116.7 (18)	O2—C5—H5	109.7
C4—N1—H1	114.8 (18)	C6—C5—H5	109.7
C2—C1—H1A	109.5	C5—C6—H6A	109.5
C2—C1—H1B	109.5	C5—C6—H6B	109.5
H1A—C1—H1B	109.5	H6A—C6—H6B	109.5
C2—C1—H1C	109.5	C5—C6—H6C	109.5
H1A—C1—H1C	109.5	H6A—C6—H6C	109.5
H1B—C1—H1C	109.5	H6B—C6—H6C	109.5
O2—C2—C1	107.42 (17)	C12—C7—C8	117.7 (2)
O2—C2—C3	109.59 (17)	C12—C7—N1	122.8 (2)
C1—C2—C3	114.0 (2)	C8—C7—N1	119.4 (2)
O2—C2—H2	108.6	C9—C8—C7	121.6 (2)
C1—C2—H2	108.6	C9—C8—H8	119.2
C3—C2—H2	108.6	C7—C8—H8	119.2
C4—C3—C2	110.3 (2)	C10—C9—C8	119.6 (2)
C4—C3—H3A	109.6	C10—C9—H9	120.2
C2—C3—H3A	109.6	C8—C9—H9	120.2
C4—C3—H3B	109.6	C9—C10—C11	119.9 (2)
C2—C3—H3B	109.6	C9—C10—Br1	119.97 (18)
H3A—C3—H3B	108.1	C11—C10—Br1	120.13 (19)
N1—C4—O1	109.21 (16)	C10—C11—C12	120.3 (2)
N1—C4—C3	113.3 (2)	C10—C11—H11	119.9
O1—C4—C3	108.37 (17)	C12—C11—H11	119.9
N1—C4—H4	108.6	C7—C12—C11	121.0 (2)
O1—C4—H4	108.6	C7—C12—H12	119.5
C3—C4—H4	108.6	C11—C12—H12	119.5
O1—C5—O2	110.92 (17)		
			
C5—O2—C2—C1	179.79 (18)	C2—O2—C5—C6	−179.48 (18)
C5—O2—C2—C3	−55.9 (2)	C4—N1—C7—C12	16.4 (3)
O2—C2—C3—C4	53.0 (2)	C4—N1—C7—C8	−166.52 (18)
C1—C2—C3—C4	173.36 (17)	C12—C7—C8—C9	−1.4 (3)
C7—N1—C4—O1	73.0 (2)	N1—C7—C8—C9	−178.59 (18)
C7—N1—C4—C3	−166.05 (17)	C7—C8—C9—C10	1.0 (3)
C5—O1—C4—N1	−176.72 (18)	C8—C9—C10—C11	0.3 (3)
C5—O1—C4—C3	59.4 (2)	C8—C9—C10—Br1	−177.60 (15)
C2—C3—C4—N1	−175.78 (17)	C9—C10—C11—C12	−1.1 (4)
C2—C3—C4—O1	−54.4 (2)	Br1—C10—C11—C12	176.77 (18)
C4—O1—C5—O2	−63.2 (2)	C8—C7—C12—C11	0.6 (3)
C4—O1—C5—C6	177.81 (19)	N1—C7—C12—C11	177.6 (2)
C2—O2—C5—O1	61.5 (2)	C10—C11—C12—C7	0.7 (4)

### Hydrogen-bond geometry (Å, º)

**Table:**

*D*—H···*A*	*D*—H	H···*A*	*D*···*A*	*D*—H···*A*
N1—H1···O1^i^	0.82 (2)	2.66 (2)	3.465 (2)	168 (2)
C8—H8···O2^i^	0.93	2.51	3.352 (3)	150
C9—H9···O2^ii^	0.93	2.65	3.557 (3)	165

Symmetry codes: (i) −*x*+1, −*y*+1, −*z*+1; (ii) *x*, *y*, *z*+1.
